# Severe Iatrogenic Facial Lipoatrophy Following Intralesional Corticosteroid Injections: Management with a Personalised Multimodal Protocol—A Case Report

**DOI:** 10.3390/reports9030261

**Published:** 2026-08-09

**Authors:** Fabrizio Melfa, Gabriele Ciranna

**Affiliations:** Mediaging Clinic Center, Private Clinic for Aesthetic and Surgical Medicine, Via Marchese di Villabianca, 90143 Palermo, Italy; dottormelfa@gmail.com

**Keywords:** iatrogenic atrophy, steroid-induced lipoatrophy, acne scar, non-ablative fractional laser, autologous regenerative therapy, calcium hydroxylapatite hyperdilution, multimodal aesthetic treatment, case report

## Abstract

**Background and Clinical Significance:** Iatrogenic atrophy following intralesional corticosteroid injections represents a rare but potentially disfiguring complication, particularly when administered in the facial region for the treatment of inflammatory acne. Unlike conventional post-acne atrophic scarring, corticosteroid-induced tissue loss involves both dermal and subcutaneous compartments, resulting in a clinically distinct presentation that poses significant therapeutic challenges, with no established consensus on optimal management. **Case Presentation:** We report the case of a 26-year-old Caucasian female (Fitzpatrick phototype III) presenting with severe iatrogenic facial atrophy of the left cheek, resulting from multiple intralesional corticosteroid injections performed by a previous physician for papulo-pustular acne. The condition had been clinically stable for approximately two years at first evaluation. The patient underwent a stepwise multimodal protocol delivered over approximately 18 months (November 2023 to April 2025), combining non-ablative fractional laser remodelling (LightScan—Eufoton), Autologous Regenerative Therapy (ART, Seffiller technique, Seffiline srl), hyperdiluted calcium hydroxylapatite (CaHA—Radiesse, Merz Aesthetics) biostimulation, and additional non-ablative fractional photothermolysis sessions, supported by a topical cosmeceutical protocol. Documented follow-up extended to December 2024, 13 months after the initiation of treatment at our centre. Clinician-assessed severity of the post-acne atrophic scarring component improved from Goodman & Baron Grade 3 to Grade 2. Global aesthetic improvement of the treated area was rated as +2 (“much improved”) on the GAIS, with a patient satisfaction score of 5/5. No validated grading instrument was applied to the subcutaneous volume deficit itself, for which no such instrument is currently available. **Conclusions:** This case documents the clinical management and favourable outcome of a patient with severe iatrogenic steroid-induced facial atrophy treated with a personalised multimodal protocol. While the observed improvement is encouraging, the single-case design does not permit conclusions regarding the efficacy, reproducibility, or generalisability of this approach. Future controlled studies are needed to determine the therapeutic value of this multimodal strategy for this rare condition.

## 1. Introduction and Clinical Significance

Intralesional corticosteroid injection is a widely used therapeutic modality in dermatology, employed for the management of inflammatory acne lesions, hypertrophic scars, and keloids [1]. When administered at appropriate doses and in the correct tissue plane, this technique is generally safe and effective. However, inadvertent delivery into the subcutaneous compartment, or the use of excessive concentrations, can result in localised dermal and subdermal atrophy—a recognised but infrequently reported iatrogenic complication [2].

Robust epidemiological data on this complication are lacking. In dermatological practice, injection-site atrophy and dyspigmentation following intralesional corticosteroid administration have been estimated to occur in approximately 0.5% of treatments [3], a figure that probably underestimates the true frequency, since mild cases resolve spontaneously and are seldom reported. Atrophy characteristically becomes clinically apparent two to four months after injection and resolves within one to two years in most cases, although persistence beyond five years has been documented [2,3]. The principal risk factors identified in the literature are the concentration and cumulative dose of the corticosteroid—a markedly increased risk of cutaneous atrophy having been reported with triamcinolone acetonide concentrations exceeding 5 mg/mL—the number and frequency of repeated injections, the use of long-acting fluorinated preparations, and the tissue plane reached by the needle: superficial dermal delivery produces predominantly epidermal and dermal thinning, whereas inadvertent delivery into the deep dermis or the subcutaneous compartment produces variable loss of adipose tissue with comparatively little epidermal change [4,5]. Thin-skinned anatomical regions, including the face, are correspondingly more susceptible [5].

Steroid-induced facial atrophy is clinically distinct from post-inflammatory atrophic acne scarring. While the latter primarily involves dermal collagen loss with characteristic morphological subtypes (ice-pick, rolling, and boxcar scars) [6], iatrogenic atrophy typically involves a broader area of tissue depression, loss of subcutaneous volume, and disruption of normal skin architecture across multiple structural layers. This compound pathology represents a significant therapeutic challenge: energy-based devices optimised for dermal remodelling may not adequately address the subcutaneous component, while volumising agents alone are insufficient to correct the textural and structural dermal deficits.

This clinical distinction is supported by histopathological and imaging evidence. In corticosteroid-induced atrophy, light, scanning and transmission electron microscopy of steroid-exposed human skin has demonstrated a marked reduction in viable epidermal thickness, flattening of the dermo-epidermal junction, and a three-dimensional reorganisation of the dermis driven by resorption of ground substance, with consequent collapse and compaction of the collagen and elastic fibre network [7]. At the molecular level, glucocorticoids directly inhibit fibroblast proliferation and suppress the synthesis of type I collagen, hyaluronic acid and other extracellular matrix components, while elastic fibres become fragmented in the superficial dermis and collapsed in the deep dermis [5,8]. Instrumental studies of skin and epidermal thickness have confirmed a measurable reduction after corticosteroid exposure, detectable before atrophy becomes clinically obvious [4]. Post-inflammatory atrophic acne scarring, by contrast, results from localised destruction and disordered repair of dermal collagen following the inflammatory lesion, producing discrete morphological subtypes within an otherwise structurally preserved dermis and an intact subcutaneous compartment [6,9]. The two processes therefore differ not only in extent but in the tissue compartments involved and in the mechanism itself: a diffuse, pharmacologically mediated matrix depletion affecting dermis and hypodermis in the first case, and a focal, inflammation-driven scarring process confined to the dermis in the second.

The absence of consensus guidelines for the management of this condition, and its relative rarity in the published literature, means that affected patients are frequently undertreated or managed sub-optimally. The present case report describes, to our knowledge, a novel structured multimodal protocol combining non-ablative fractional laser remodelling, Autologous Regenerative Therapy (ART), and hyperdiluted calcium hydroxylapatite biostimulation, achieving sustained clinical improvement in a young patient with severe, stabilised iatrogenic facial atrophy.

## 2. Case Presentation

### 2.1. Patient History

A 26-year-old Caucasian female (Fitzpatrick phototype III) presented to our clinic complaining of severe left facial depression and skin texture abnormality, with significant psychological and aesthetic impact. The patient reported a history of moderate-to-severe papulo-pustular acne of the left cheek, for which she had received multiple intralesional corticosteroid injections administered by a previous physician. The patient was unable to provide the specific corticosteroid formulation, concentration, number of injections, injected volumes, or precise injection sites and tissue plane. She reported that visible tissue depression developed progressively over the months following the injection course. No clinical records from the treating physician were available for review. Following these injections, she developed a progressive and eventually stable area of significant tissue atrophy involving the left cheek, characterised by both dermal and subcutaneous loss, which she described as “a scar and important loss of tissue.”

The atrophic defect had been clinically stable for approximately two years prior to her first evaluation at our centre. The patient denied any subsequent acne activity, current pharmacological therapy, or known drug allergies. She had previously received systemic isotretinoin therapy, with the last course completed in 2020. No hormonal therapy, prior surgical intervention to the area, or prior laser or energy-based device treatments to the left cheek were reported.

### 2.2. Clinical Examination

Examination of the left cheek revealed two anatomically distinct but coexisting pathological components:

Superior component (mid-cheek): Multiple atrophic scars with mixed ice-pick and rolling morphology distributed over an extended surface area, associated with chromatic heterogeneity and erythematous discolouration—consistent with residual post-acne dermal scarring.

Inferior–central component (lower cheek): A broad area of significant dermal and subcutaneous atrophy with tissue depression, irregular surface contour, and partial erythema—consistent with iatrogenic corticosteroid-induced lipoatrophy, anatomically and morphologically distinct from typical acne-related scarring.

Baseline severity of the atrophic acne scarring component (superior mid-cheek area) was graded as Grade 3 (moderate) on the Goodman & Baron Qualitative Grading System [6], indicating lesions that were clearly visible at conversational distance, not fully concealable by make-up, with mixed rolling and ice-pick morphology across an estimated surface area of 6–8 cm^2^. Formal GAIS assessment—which quantifies improvement relative to a documented baseline rather than absolute severity—was performed at each subsequent follow-up visit and is reported in Section 2.4.

The superior component involved multiple atrophic scars of mixed rolling and ice-pick morphology, distributed over an estimated surface area of approximately 6–8 cm^2^ (approximately 3 cm in vertical height × 2–2.5 cm in width), with chromatic heterogeneity, areas of residual erythema, and focal hypopigmentation. Surface texture was irregular with partial loss of normal follicular architecture. The inferior component presented as a broad area of compound dermal and subcutaneous atrophy, estimated at 8–12 cm^2^ in surface extent (approximately 4 cm × 2.5–3 cm), with a characteristic crinkled-paper surface texture (crinkled paper sign), focal telangiectasia, and visible tissue depression consistent with iatrogenic corticosteroid-induced lipoatrophy. However, no objective volumetric, ultrasonographic, or histopathological assessment was performed to confirm the depth and extent of subcutaneous involvement, and these findings should be interpreted as clinical impressions rather than instrumentally verified diagnoses. Precise dimensional measurements were not recorded at the initial 2022 assessment; clinical estimation is based on photographic documentation. Standardised photographic documentation was initiated with VISIA^®^ Complexion Analysis [10] from February 2024 onwards.

**Figure 1:**
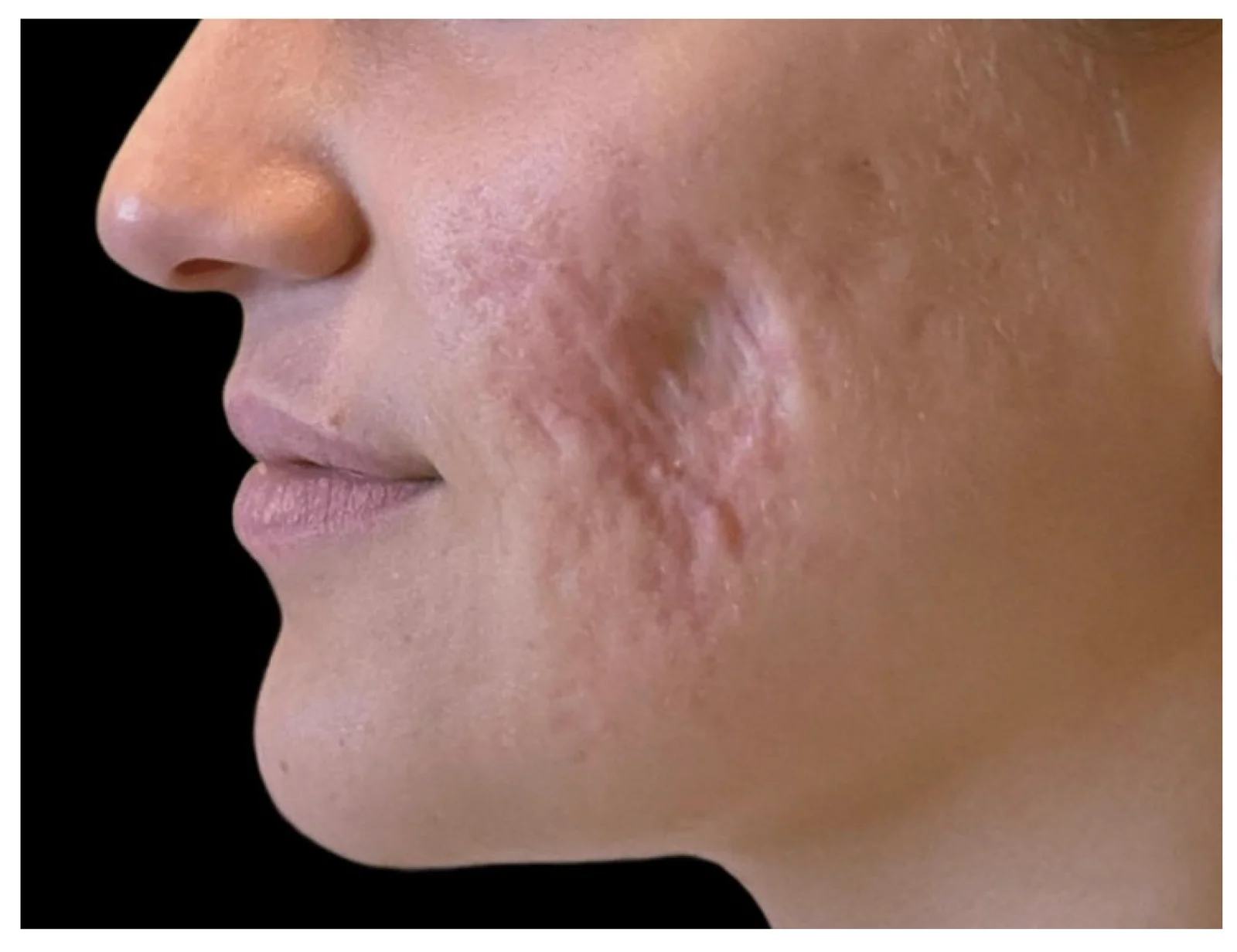
Clinical photographs at baseline (T0, October 2022), obtained with a standard smartphone camera at the referring centre in Bologna. Left lateral view demonstrating the extent of iatrogenic corticosteroid-induced atrophic defect of the left cheek, with compound dermal and subcutaneous tissue loss. No standardised imaging system was available at the time of initial evaluation.

Photographic documentation of the baseline condition (T0) was obtained at the external centre in Bologna in October 2022 using a standard smartphone camera (Figure 1). No standardised complexion analysis instrumentation (VISIA^®^ or equivalent) was available at that centre. This represents an inherent limitation in the pre-treatment baseline documentation, as discussed in Section 3. VISIA^®^ Complexion Analysis System (Canfield Scientific, Parsippany, NJ, USA) imaging was performed from February 2024 onwards, providing standardised photographic documentation for all subsequent visual comparisons.

### 2.3. Therapeutic Protocol

A stepwise multimodal protocol was designed with the dual objective of (i) stimulating neocollagenesis and structural dermal regeneration, and (ii) restoring the lost subcutaneous volume, while progressively improving the surface texture of the atrophic component. The sequence of treatments was planned with the intent of tissue preparation and remodelling preceding volume restoration, followed by surface refinement with non-ablative fractional photothermolysis. The complete treatment timeline is summarised in Table 1.

#### 2.3.1. Non-Ablative Fractional Laser Treatment (LightScan—Eufoton) (Sessions 1–4)

In this protocol, a non-ablative fractional laser device (LightSCAN™, Eufoton S.r.l., Trieste, Italy) was used in its dedicated fractional handpiece modality, operating at 1470 nm. This handpiece delivers controlled fractional energy to the dermis through a scanning mechanism, generating an array of microscopic thermal columns with dermal remodelling and neocollagenesis-stimulating effects, without ablation of the epidermal surface. This approach was selected for its suitability in treating the superficial and mid-dermal components of the atrophic defect, including the scar borders, where stimulation of the perilesional tissue was a specific therapeutic objective.

Four sessions were performed (November 2023, February 2024, September 2024, and April 2025), with progressive energy adjustment reflecting the evolving tissue response:

The fractional handpiece was applied using the “acne scarring” preset protocol at 20% density, as per the manufacturer’s established parameters for atrophic scar treatment. The technique involved rotating the handpiece over the treatment zone in a systematic scanning pattern, with approximately 4–5 passes per side across the full facial area treated. In the zone corresponding to the cicatricial defect, additional passes were performed relative to the contralateral side, with deliberate treatment of the scar border as well as the central atrophic area in order to stimulate the perilesional tissue and promote progressive scar margin blending. The energy parameters delivered across the four sessions were as follows: Session 1 (November 2023): 5.5 W/660 J; Session 2 (February 2024): 5.5 W/1000 J; Session 3 (September 2024): 5.5 W/600 J; and Session 4 (April 2025): 4.5 W/450 J. The progressive increase in energy between Sessions 1 and 2 reflects deliberate escalation in response to satisfactory tissue tolerance, while the reduction in Sessions 3 and 4 reflects a maintenance strategy in a tissue environment already substantially remodelled by the intervening procedures.

All sessions were performed under topical anaesthesia with 20% lidocaine cream, applied to the treatment area prior to each session. Immediately following treatment, Betamethasone valerate 0.1% + Gentamicin sulphate 0.1% was applied to the treated surface as an anti-inflammatory and antimicrobial prophylactic measure. No additional post-procedural dressings or occlusive protocols were employed. No intra-procedural or post-procedural complications were recorded across any of the four sessions.

#### 2.3.2. Autologous Regenerative Therapy (April 2024)

Autologous Regenerative Therapy (ART) is a technique for the preparation and injection of an autologous stromal vascular fraction (SVF)-enriched lipograft, obtained through mechanical emulsification of harvested adipose tissue without enzymatic processing (Seffiller^®^ technique, Seffiline S.r.l., Bologna, Italy). The resulting product is characterised by a high concentration of adipose-derived stromal cells (ADSCs), growth factors, and extracellular matrix components, with documented capacity to promote angiogenesis, tissue regeneration, and collagen synthesis in atrophic and scarred tissue beds [11].

A total volume of 18 mL of autologous adipose-derived cells was prepared according to the ART protocol and injected into the atrophic zone of the left cheek. Injection was performed using a 21-gauge cannula. A double-plane technique was employed, with product delivered into both the subcutaneous and the deeper subdermal plane, targeting the full volume deficit of the atrophic defect. Treatment was distributed across both the peripheral and the central areas of the lesion in order to achieve uniform volumetric restoration and tissue integration. No local anaesthesia was required, as the autologous fat preparation itself provides intrinsic analgesic effect.

#### 2.3.3. CaHA Hyperdiluted 1:2 (September 2024)

Calcium hydroxylapatite (CaHA; Radiesse^®^, Merz Aesthetics, Merz North America, Inc., Raleigh, NC, USA), administered in a hyperdiluted formulation, has established biostimulatory properties, promoting fibroblast activation and de novo collagen and elastin synthesis in the injected tissue plane without the volumising effect of standard-concentration preparations [12,13]. The technique was employed to deliver the product in a highly diluted form, thereby achieving a diffuse biostimulatory effect across the atrophic zone.

A 1:2 hyperdilution of CaHA was prepared as follows: 1.5 mL of CaHA (one full vial) was combined with 2.7 mL of normal saline (0.9% NaCl) (Salf Laboratorio Farmacologico, Bergamo, Italy) and 0.3 mL of lidocaine (Galenic Laboratory of Farmacia Metalla, Milano, Italy), yielding a total volume of 4.5 mL of the hyperdiluted product. The entire prepared volume was injected into the left cheek atrophic zone using a 25-gauge cannula (TSK STERiGLIDE, TSK Laboratory, Tochigi, Japan) in a subdermal plane. Local anaesthesia was administered prior to injection. The technique involved retrograde linear threading with fan-pattern distribution to achieve homogeneous coverage of the treatment area.

#### 2.3.4. Non-Ablative Fractional Photothermolysis 1470 nm (September 2024)

An additional non-ablative fractional laser session was performed in combination with CaHA in September 2024, using the same device, handpiece, and preset described in Section 2.3.1 (5.5 W/600 J). The laser was applied immediately before CaHA delivery to precondition the dermal compartment [9,14].

#### 2.3.5. Cosmeceutical Support Protocol

Throughout the treatment course, the patient was maintained on a structured topical cosmeceutical protocol comprising a pharmaceutical-grade retinol preparation (ZO Skin Health^®^, ZO Skin Health, Inc., Irvine, CA, USA) in combination with a stabilised topical 10% vitamin C serum (ZO Skin Health^®^, ZO Skin Health, Inc., Irvine, CA, USA). The retinol component was employed to prime the skin between sessions, upregulate fibroblast activity, and promote ongoing collagen synthesis, while topical vitamin C provided antioxidant support, enhanced collagen cross-linking, and contributed to progressive improvement in skin chromatic homogeneity. Broad-spectrum photoprotection (SPF 50+) was mandatory throughout the entire treatment period.

### 2.4. Outcome and Follow-Up

Clinical outcome was assessed at each follow-up visit through standardised clinical photography, VISIA^®^ Complexion Analysis imaging (from February 2024 onwards), patient-reported outcome measures using a five-point satisfaction scale, clinician-rated scar severity using the Goodman & Baron Qualitative Scale, and global improvement relative to baseline using the GAIS. The outcome data across all time points are summarised in Table 2.

The Goodman & Baron Qualitative Grading System was applied exclusively to the post-acne atrophic scarring component of the superior mid-cheek area, for which it was developed and validated. No validated grading instrument was applied to the steroid-induced subcutaneous volume deficit of the inferior component, as no scale validated for facial lipoatrophy of iatrogenic origin is currently available; the response of this component was documented by standardised photography and is reflected only indirectly in the GAIS and patient satisfaction ratings, which refer to the treated area as a whole.

**Figure 2:**
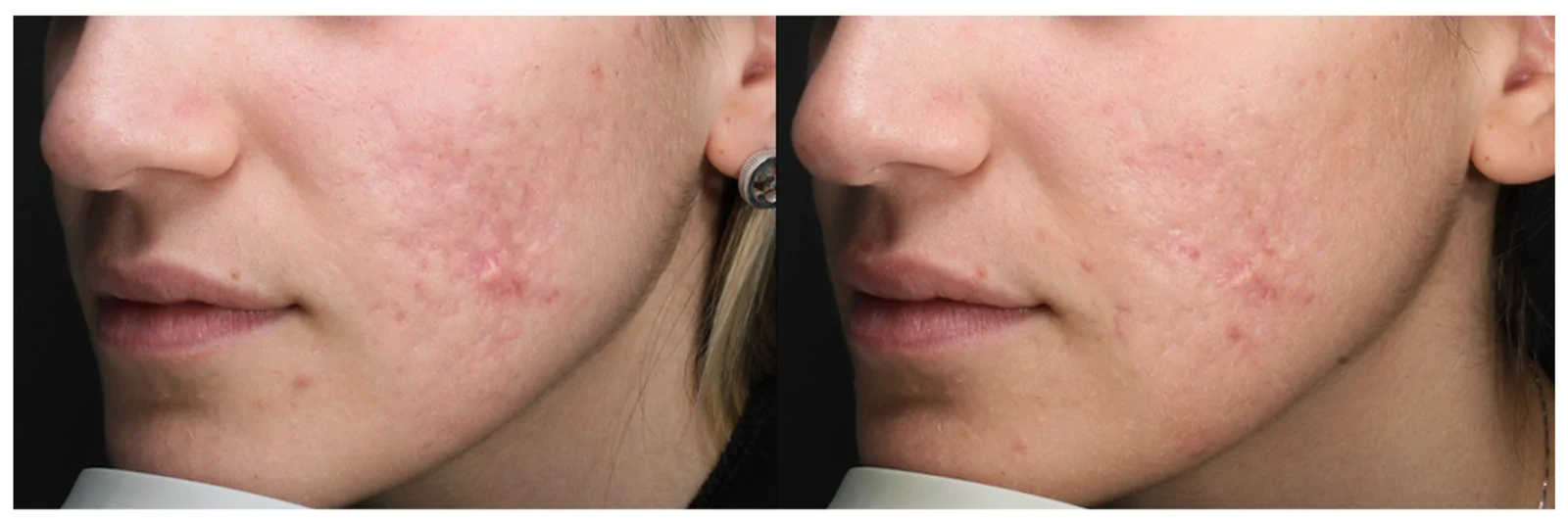
VISIA^®^ Complexion Analysis composite images of the left cheek, standardised lighting and positioning. **Left panel**: baseline assessment (February 2024), showing diffuse erythema overlying the atrophic scar complex, with marked chromatic contrast between the lesional and perilesional skin and an irregular surface texture. **Right panel**: follow-up assessment (September 2024), demonstrating reduced erythema, improved chromatic homogeneity between the scarred and surrounding skin, and a smoother overall surface texture, consistent with the progressive biostimulatory effect of the treatment protocol.

**Figure 3:**
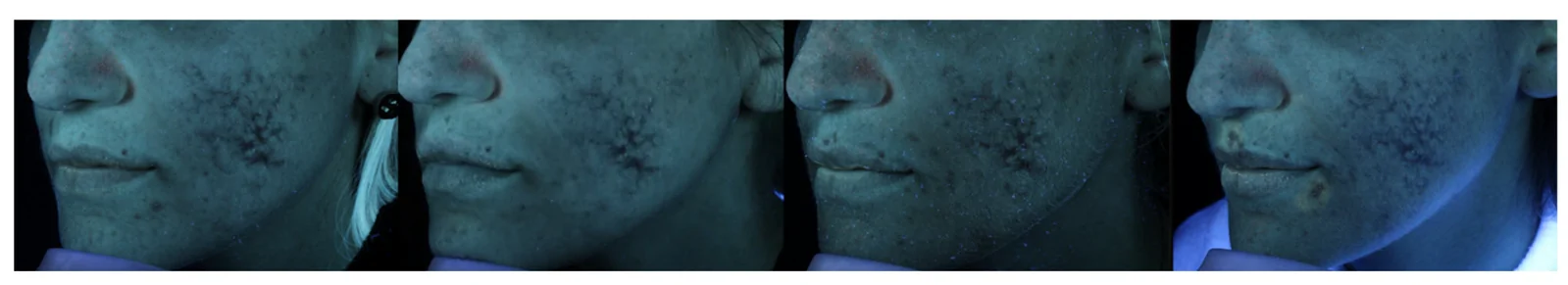
Sequential VISIA^®^ UV fluorescence imaging of the left cheek across four standardised photographic documentation time points, arranged left to right in chronological order: T1 (16 February 2024), T2 (22 April 2024), T3 (30 September 2024), and T4 (1 December 2024). UV fluorescence highlights porphyrin activity and subclinical changes not visible under standard lighting, providing complementary documentation of the progressive improvement in skin quality over the treatment course.

**Figure 4:**
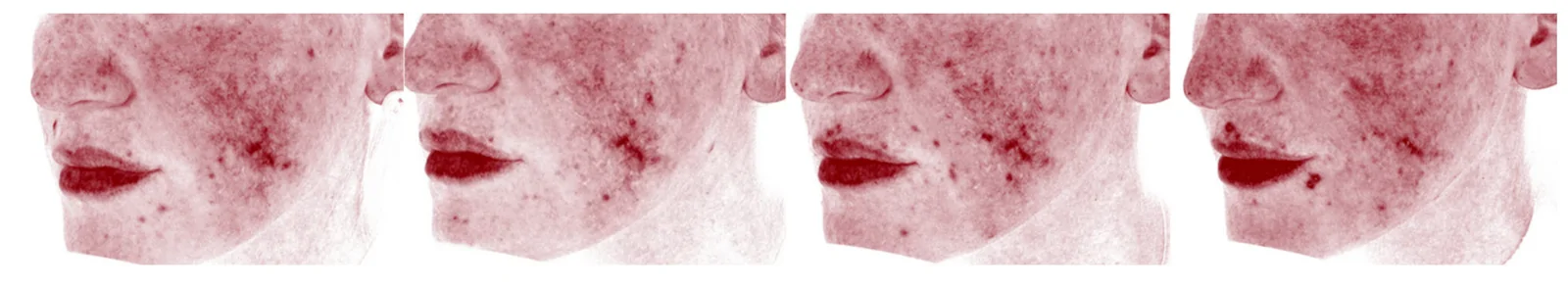
Sequential VISIA^®^ haemoglobin/vascularity (red areas) imaging of the left cheek across four standardised photographic documentation time points, arranged left to right in chronological order: T1 (16 February 2024), T2 (22 April 2024), T3 (30 September 2024), and T4 (1 December 2024). This modality isolates subsurface haemoglobin signal to visualise erythema and vascular components not fully apparent under standard lighting, complementing the texture and UV fluorescence assessments shown in Figure 2 and Figure 3.

Serial VISIA^®^ acquisitions, performed at the first standardised photographic documentation session (February 2024) and at subsequent visits (April 2024, September 2024, December 2024), demonstrated progressive improvement in surface texture and chromatic homogeneity on visual comparison of standardised photographic documentation. Representative VISIA^®^ composite images are presented in Figure 2. In addition to standard-light complexion analysis, UV fluorescence and haemoglobin/vascularity (red areas) imaging were performed at each VISIA^®^ session to assess porphyrin activity, subclinical pigmentary changes, and erythema/vascular components not fully apparent under standard lighting, providing complementary standardised photographic documentation of the treatment response across all time points (Figure 3 and Figure 4).

**Figure 5:**
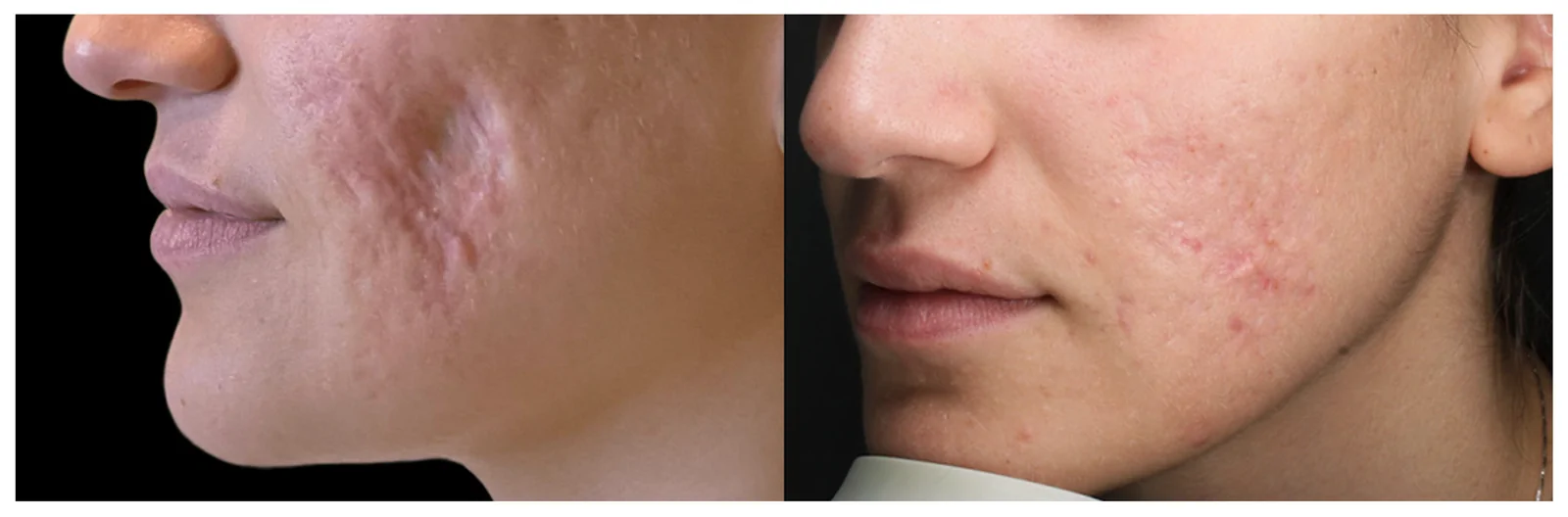
Comparative clinical photographs of the left cheek. **Left panel**: baseline (T0, October 2022), obtained with a standard smartphone camera at the referring centre in Bologna, showing the full extent of the iatrogenic corticosteroid-induced atrophic defect with the characteristic linear atrophic/depressed scar component consistent with iatrogenic lipoatrophy and overlying acneiform atrophic scarring. **Right panel**: 13-month follow-up (December 2024), VISIA^®^ Complexion Analysis System acquisition, demonstrating substantial improvement of the atrophic defect, marked improvement in surface texture and chromatic homogeneity, with only minimal residual scarring evident.

No post-procedural complications were recorded across any of the treatment sessions. The patient reported no adverse events between sessions and expressed high overall satisfaction with the aesthetic outcome. At final follow-up, clinician-assessed scar severity had improved to Goodman & Baron Grade 2. Global aesthetic improvement relative to baseline was rated as +2 (“much improved”) on the GAIS. Patient-reported satisfaction at final follow-up was 5/5. The clinical outcome reported in this manuscript was assessed at the December 2024 time point, approximately 13 months after the initiation of treatment at our centre (November 2023). The treatment protocol itself extended over approximately 18 months, to the final maintenance session of April 2025; however, no formal outcome assessment was conducted at that time point, and the December 2024 evaluation represents the most recent documented assessment of treatment response (Figure 5). Documented follow-up therefore covers 13 months and is to be distinguished from the 18-month duration of the treatment protocol.

## 3. Discussion

The present case illustrates the clinical and therapeutic complexity of iatrogenic corticosteroid-induced facial atrophy—a condition that, despite being a recognised complication of intralesional steroid therapy, remains inadequately characterised in the aesthetic medicine literature and lacks established management guidelines.

The key pathophysiological distinction between steroid-induced lipoatrophy and conventional post-acne atrophic scarring has direct therapeutic implications. In common post-acne scarring, the primary target is dermal collagen remodelling, and fractional laser, microneedling, and subcision protocols address this effectively [15,16]. In contrast, corticosteroid-induced atrophy involves a compound deficit: not only dermal structural loss, but also subcutaneous tissue dissolution and fibrotic disruption of the dermo-hypodermal junction. This necessitates a multilayer therapeutic approach that addresses the subcutaneous compartment as a primary target, with surface refinement as a secondary objective.

The therapeutic sequence adopted in this case was designed with this rationale in mind. Non-ablative fractional laser treatment was employed as the foundational intervention: by delivering fractional 1470 nm energy transcutaneously via the dedicated scanning handpiece, it initiates a fibroblastic wound-healing response in the dermis and at the dermo-hypodermal interface, creating a progressively remodelled tissue environment favourable to the subsequent volumising and regenerative procedures. The choice of a non-ablative fractional laser (1470 nm) over an ablative modality (e.g., fractional CO_2_) was clinically motivated: the primary pathology involves predominantly subcutaneous and deep dermal tissue loss rather than superficial epidermal irregularity; the overlying skin retained sufficient structural integrity to serve as a biological scaffold for the subsequent regenerative and biostimulatory interventions, which ablative treatment would have disrupted; and the non-ablative wavelength was selected for its capacity to induce controlled dermal remodelling at the dermo-hypodermal interface without ablating the epidermal surface, thereby creating a thermally preconditioned tissue bed receptive to the biological agents applied subsequently. Of particular note was the deliberate treatment of the scar borders in addition to the central atrophic area in order to stimulate perilesional tissue and promote gradual integration of the healthy surrounding dermis with the atrophic zone. The progressive adjustment of energy parameters across sessions (660 J, 1000 J, 600 J, 450 J) reflects a deliberate clinical strategy of tissue conditioning followed by consolidation, adapted to the evolving tissue response.

ART was selected primarily for its regenerative biological activity rather than for simple volumetric filling. In iatrogenic steroid-induced lipoatrophy, the tissue loss is accompanied by disruption of the extracellular matrix architecture, vascular compromise, and fibrotic changes that are not addressed by volumetric filling alone. ART was selected for its unique biological composition: as a mechanically processed autologous lipograft enriched in ADSCs and growth factors, it provides both volumetric restoration and regenerative biological activity, without the risks associated with enzymatic SVF preparation or the limitations of cell-depleted fat grafting. Its application in a tissue bed pre-conditioned by non-ablative fractional laser thermal treatment may potentiate graft survival and biological efficacy through enhanced angiogenesis and tissue receptivity.

The biological rationale for ART in this setting rests on the composition of the graft rather than on its volume. Mechanical emulsification without enzymatic digestion yields a lipoaspirate enriched in adipose-derived stromal cells, pericytes and endothelial progenitors, together with the growth factors and extracellular matrix components of the stromal vascular fraction [11]. These cells act principally through paracrine signalling: secretion of vascular endothelial growth factor and basic fibroblast growth factor promotes neoangiogenesis in the recipient bed, while a subpopulation differentiates into adipocytes and contributes to durable volume restoration. This is directly pertinent to corticosteroid-induced lipoatrophy, in which the pharmacological insult has produced precisely the deficits that these mechanisms address—suppressed fibroblast activity, depleted extracellular matrix and loss of adipocyte mass [5,8]. Clinical support comes from stromal-cell-enriched fat grafting in facial lipoatrophy of other aetiologies: Yoshimura and colleagues reported that supplementation of the graft with adipose-derived stem cells (cell-assisted lipotransfer) in patients with facial lipoatrophy secondary to lupus profundus and Parry–Romberg syndrome produced superior and more durable contour restoration than conventional lipoinjection [17]. Direct clinical evidence in corticosteroid-induced facial lipoatrophy specifically is, to our knowledge, limited to isolated reports; the extrapolation from these related conditions is therefore an assumption underlying the present protocol rather than an established indication.

Hyperdiluted CaHA was subsequently introduced as a biostimulatory agent targeting the dermal–subdermal interface [18,19]. In its hyperdiluted form, CaHA acts primarily as a scaffold and stimulus for endogenous collagen and elastin synthesis, complementing the regenerative activity of ART and contributing to progressive structural improvement of the atrophic zone.

Non-ablative fractional photothermolysis (1470 nm) was performed in the same session as CaHA: fractional laser-induced microcoagulation activates a controlled wound-healing cascade in the dermis, while the simultaneously administered CaHA provides a structural biostimulatory substrate for the ensuing neocollagenesis [20]. It is not possible to determine from this single case whether the combination of these two modalities in a single session conferred any advantage over their sequential application in separate sessions.

A recent case report described the successful treatment of a single facial steroid-induced atrophic scar using a dual-session fractional CO_2_ laser protocol preceded by polydioxanone thread placement and a dimethylaminoethanol skin booster, with VSS improving from 9/13 at baseline to 2/13 at the final follow-up [21]. This confirms the relevance of fractional laser-based dermal remodelling for this condition and supports the choice of fractional photothermolysis as a core component of the present protocol. However, that case addressed a recent (approximately one-month-old), single-component atrophic defect using a laser-only approach with adjunctive mechanical and biostimulatory priming, whereas the present case involved a long-stabilised (approximately two-year), compound dermal and subcutaneous defect requiring an additional volumetric and regenerative component (ART and hyperdiluted CaHA) beyond laser-induced dermal remodelling alone. This distinction underscores that the optimal therapeutic strategy for steroid-induced atrophy may depend substantially on lesion chronicity and the relative contribution of subcutaneous volume loss, supporting the rationale for a multimodal rather than single-modality approach in cases with established subcutaneous involvement. This comparison is itself limited. It rests on a single published case treated with a different protocol, assessed with different instruments and reported by different observers, and no formal comparison between the two approaches is possible. It is offered as a contextual observation on the range of strategies described for this rare condition, not as evidence that either approach is superior to the other.

The selection of ART and hyperdiluted CaHA as core components of this protocol was informed by the senior author’s prior clinical experience with these techniques [22,23,24], as well as by independent evidence supporting the regenerative capacity of adipose-derived stromal cells in tissue repair [11] and the biostimulatory efficacy of hyperdiluted CaHA in dermal remodelling [13,19]. The present case applies this combined SEFFI/CaHA rationale to a more complex clinical scenario—a compound, long-stabilised iatrogenic defect—integrated with non-ablative fractional laser remodelling.

The contribution of the topical cosmeceutical protocol to the observed improvement cannot be quantified, but it is biologically plausible and merits explicit consideration. Retinol is of particular interest in this specific context: topical all-trans-retinoic acid has been experimentally shown to prevent corticosteroid-induced skin atrophy without abrogating the anti-inflammatory activity of the steroid [25], acting through upregulation of fibroblast activity and modulation of collagen, glycosaminoglycan and fibronectin synthesis—precisely the pathways suppressed by glucocorticoid exposure [5,8]. Retinoids may therefore represent a rational adjunct in the management of steroid-induced atrophy rather than a generic measure of skin-quality maintenance. Topical ascorbic acid acts as an essential cofactor for prolyl and lysyl hydroxylase, the enzymes responsible for the post-translational hydroxylation that stabilises the collagen triple helix, and additionally provides antioxidant protection and inhibits tyrosinase, which is relevant to the chromatic heterogeneity observed in this patient [26]. Broad-spectrum photoprotection, maintained throughout the treatment course, limits ultraviolet-induced matrix metalloproteinase activation and post-inflammatory hyperpigmentation in skin repeatedly subjected to fractional thermal injury. These agents were applied continuously and concurrently with the procedural interventions; their individual contribution is therefore inseparable from that of the laser, ART and CaHA, and the possibility that a non-trivial part of the observed textural and chromatic improvement is attributable to the cosmeceutical protocol alone cannot be excluded.

Although the individual contribution of each modality cannot be determined from a single case, the intended role of each within the protocol can be stated explicitly. The non-ablative fractional laser was directed at the dermal component and is the modality whose action would most plausibly account for the improvement in surface texture and in the appearance of the atrophic acne scars of the superior area, consistent with the established evidence for fractional photothermolysis in atrophic scarring [14,15]. ART was directed at the subcutaneous deficit and is the only component of the protocol that delivers volume, so that the restoration of contour in the depressed inferior area is most plausibly attributable to it. Hyperdiluted CaHA was directed at the dermal–subdermal interface, where its biostimulatory action would be expected to contribute to progressive firmness and to the consolidation of dermal quality over the months following injection [13,19]. The cosmeceutical protocol operated continuously in the background throughout. This mapping of modality to target is a statement of therapeutic intent and of mechanistic plausibility, not a measurement of effect: the temporal sequence of the observations is equally compatible with alternative attributions and with a cumulative effect to which every component contributed to an unknown degree.

**Figure 6:**
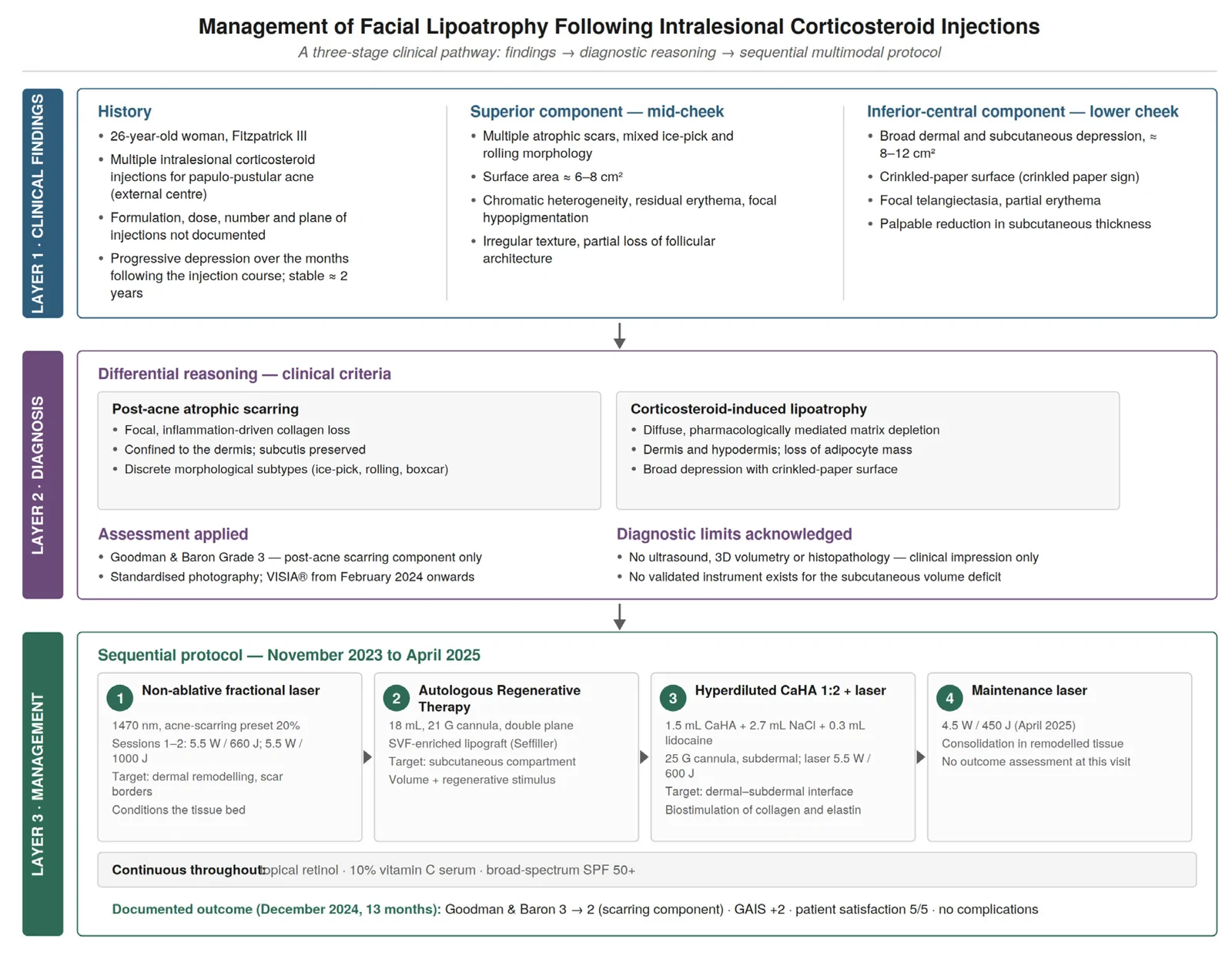
Three-layer schematic of the clinical pathway followed in this case. Layer 1 summarises the clinical findings, including the history of intralesional corticosteroid injections and the two anatomically distinct components identified on examination. Layer 2 sets out the diagnostic reasoning by which post-acne atrophic scarring was distinguished from corticosteroid-induced lipoatrophy, the assessment instruments applied, and the diagnostic limitations acknowledged. Layer 3 presents the sequential multimodal protocol, the target compartment of each modality, the continuous cosmeceutical support, and the outcome documented at the December 2024 assessment. The figure describes the pathway followed in this single case and is not proposed as a validated treatment algorithm.

Standardised VISIA^®^ photographic documentation was only initiated in February 2024—approximately 15 months after the condition had already been present and photographically documented. This gap prevents standardised photographic comparison between the true baseline condition and the final outcome, and represents an acknowledged methodological limitation of this report. Additionally, the long-term durability of the achieved results beyond the documented follow-up period of 13 months (November 2023 to December 2024) remains to be determined. Furthermore, outcome assessment relied predominantly on clinician-rated qualitative scales (Goodman & Baron, GAIS) and patient-reported satisfaction, without quantitative instrumental measurements. The Goodman & Baron scale, moreover, is validated for post-acne atrophic scarring and was applied only to that component: no validated instrument was applied to the subcutaneous volume deficit that constitutes the principal subject of this report, and the response of that component is therefore documented photographically and through global scales rather than through a dedicated validated measure. We wish to emphasise that the present case report is hypothesis-generating in nature and does not constitute scientific validation of the described protocol. The therapeutic approach was an individualised clinical decision, informed by the senior author’s prior experience and the available literature, but representing a personalised adaptation rather than a standardised treatment algorithm. In the absence of controlled comparative data, it is not possible to attribute the observed clinical improvement to any single modality, to the specific combination employed, or to the sequential order of interventions. It is also not possible to determine whether the combination conferred any advantage over individual modalities applied independently. The favourable outcome reported here should therefore be interpreted as preliminary clinical evidence supporting the potential rationale for a multimodal approach to this condition, rather than as proof of therapeutic efficacy. This case has several limitations inherent to its nature as a single-patient report. The primary limitation concerns baseline documentation: the initial clinical assessment in October 2022 was performed at an external centre in Bologna where no standardised complexion analysis instrumentation was available. Photographic documentation at T0 was therefore limited to smartphone photography, without standardised lighting, fixed focal length, or instrumental analysis. A significant limitation is the absence of detailed information regarding the causative corticosteroid injections (formulation, concentration, number, volume, and injection plane), which were administered by a previous physician and reported by the patient without supporting clinical documentation. The distinction between the post-acne atrophic scar component and the steroid-induced lipoatrophy component was based on clinical assessment alone, without objective confirmation by ultrasound, three-dimensional volumetric imaging, or histopathological examination. An additional limitation is the absence of a dedicated post-treatment follow-up assessment after the final maintenance session (April 2025); the outcome data reported correspond to the December 2024 evaluation. A prospective series with standardised outcome measures, objective diagnostic imaging, and extended follow-up would be required to determine the therapeutic value of this approach. The clinical findings, the diagnostic reasoning and the sequential protocol described above are summarised schematically in Figure 6.

## 4. Conclusions

This case report documents the clinical management and favourable outcome of a patient with severe, long-stabilised iatrogenic facial lipoatrophy treated with a personalised multimodal protocol combining non-ablative fractional laser remodelling, Autologous Regenerative Therapy (ART), hyperdiluted CaHA biostimulation, and additional non-ablative fractional photothermolysis sessions, supported by a cosmeceutical protocol. While the observed improvement is encouraging, the single-case design does not permit conclusions regarding the efficacy, reproducibility, or generalisability of this approach.

The protocol described represents an individualised clinical decision informed by the senior author’s prior published experience [22,23,24] and should not be interpreted as a validated treatment algorithm. The detailed description of the treatment course, clinical reasoning, and observed outcome is offered as a contribution to the limited literature on this rare condition, and may inform future clinical decision-making in similar cases. Further controlled studies with larger cohorts, standardised outcome measures, objective diagnostic imaging, and extended follow-up are needed to determine whether this multimodal strategy offers genuine therapeutic advantages over single-modality approaches for this rare and therapeutically challenging condition.

## Figures and Tables

**Table 1:** Chronological summary of all treatment sessions.

Date	Location	Procedure	Parameters/Notes
20 October 2022	Bologna	Clinical evaluation + Videodermatoscopy	T0 photo documentation with smartphone. No Visia available. Unique pre-treatment photographic baseline.
	Home-based	At-home facial treatment	Following the initial consultation, I began a treatment programme using ZO Skin Health products, comprising a facial cleanser, a scrub and 1% retinol (Wrinkle & Texture Repair, ZO Skin Health, Inc., Irvine, CA, USA)
8 November 2023	Palermo	Non-ablative fractional laser—Session 1	Scarring Acne Preset 20%—non-ablative fractional handpiece, 5.5 W/660 J total. Anaesthesia: lidocaine cream 20% (Galenic Laboratory of Farmacia Metalla, Milano, Italy). Post-proc: Gentamicin and Betamethasone.
16 February 2024	Palermo	Non-ablative fractional laser—Session 2	Scarring Acne Preset 20%—non-ablative fractional handpiece, 5.5 W/1000 J total. Anaesthesia: lidocaine cream 20%. Post-proc: Gentamicin and Betamethasone.
22 April 2024	Palermo	Autologous Regenerative Therapy (ART)	18 mL autologous fat cells. Cannula 21 G (TSK STERiGLIDE, TSK Laboratory, Tochigi, Japan). Double plane (subcutaneous + deep). Central and peripheral distribution. Integrated anaesthesia of fat.
30 September 2024	Palermo	CaHA + non-ablative fractional laser—Session 3	CaHA 1.5 mL + 2.7 mL NaCl + 0.3 mL lidocaine (1:2 dilution); 25 G cannula, subdermal plane. Non-ablative fractional laser (LightScan—Eufoton): Scarring Acne Preset 20%, 5.5 W/600 J total, same session.
9 April 2025	Palermo	Non-ablative fractional laser—Session 4	Scarring Acne Preset 20%—non-ablative fractional handpiece, 4.5 W/450 J total (maintenance session). Anaesthesia: lidocaine cream 20%. Post-proc: Gentamicin and Betamethasone.

**Table 2:** Clinical outcomes across the treatment timeline.

Time Point	Date	Goodman & Baron	GAIS	Satisf. (/5)
T0	October 2022	Grade 3	—	0/5
T1 (1st laser)	November 2023	Grade 3	+1	1/5
T2 (2nd laser)	February 2024	Grade 3	+1	1/5
T3 (ART)	April 2024	Grade 2–3	+1	3/5
T4 (CaHA + 3rd laser)	September 2024	Grade 2	+2	4/5
T5—final documented assessment	December 2024	Grade 2	+2 (much improved)	5/5
4th laser (maintenance treatment session; no outcome assessment performed)	April 2025	—	—	—

Goodman & Baron = Goodman & Baron Qualitative Grading Scale, applied to the post-acne atrophic scarring component of the superior mid-cheek area only; Abbreviations: GAIS = Global Aesthetic Improvement Scale (+1 improved, +2 much improved, +3 very much improved), referring to the treated area as a whole; Satisf. = patient-reported satisfaction (1–5). The April 2025 session was a maintenance treatment; no formal outcome assessment was performed at that time point, and the December 2024 evaluation represents the most recent documented assessment of treatment response.

## Data Availability

The data presented in this study are available on request from the corresponding author due to patient privacy considerations.

## References

[1] Richards R.N. (2010). Update on intralesional steroid: Focus on dermatoses. J. Cutan. Med. Surg..

[2] Colwell R., Gullickson M., Cutlan J., Stratman E. (2025). Cutaneous Atrophy Following Corticosteroid Injections for Tendonitis: Report of Two Cases. JMIR Dermatol..

[3] Dhinsa H., McGuinness A.E., Ferguson N.N. (2021). Successful treatment of corticosteroid-induced cutaneous atrophy and dyspigmentation with intralesional saline in the setting of keloids. JAAD Case Rep..

[4] Barnes L., Kaya G., Rollason V. (2015). Topical corticosteroid-induced skin atrophy: A comprehensive review. Drug Saf..

[5] Niculet E., Bobeica C., Tatu A.L. (2020). Glucocorticoid-induced skin atrophy: The old and the new. Clin. Cosmet. Investig. Dermatol..

[6] Goodman G.J., Baron J.A. (2006). Postacne scarring: A qualitative global scarring grading system. Dermatol. Surg..

[7] Lehmann P., Zheng P., Lavker R.M., Kligman A.M. (1983). Corticosteroid atrophy in human skin. A study by light, scanning, and transmission electron microscopy. J. Investig. Dermatol..

[8] Schoepe S., Schäcke H., May E., Asadullah K. (2006). Glucocorticoid therapy-induced skin atrophy. Exp. Dermatol..

[9] Gozali M.V., Zhou B. (2015). Effective treatments of atrophic acne scars. J. Clin. Aesthetic Dermatol..

[10] Henseler H. (2022). Validation of the Visia^®^ Camera System for skin analysis through assessment of the correlations among the three offered measurements—The percentile, feature count and absolute score—As well as the three capture perspectives, from the left, front and right. GMS Interdiscip. Plast. Reconstr. Surg. DGPW.

[11] Rossi M., Roda B., Zia S., Vigliotta I., Zannini C., Alviano F., Bonsi L., Zattoni A., Reschiglian P., Gennai A. (2020). Characterization of the Tissue and Stromal Cell Components of Micro-Superficial Enhanced Fluid Fat Injection (Micro-SEFFI) for Facial Aging Treatment. Aesthetic Surg. J..

[12] Muti G.F., Astolfi G., Renzi M., Rovatti P.P. (2015). Calcium Hydroxylapatite for Augmentation of Face and Hands: A Retrospective Analysis in Italian Subjects. J. Drugs Dermatol..

[13] Rovatti P.P., Pellacani G., Guida S. (2020). Hyperdiluted Calcium Hydroxylapatite 1: 2 for Mid and Lower Facial Skin Rejuvenation: Efficacy and Safety. Dermatol. Surg..

[14] Hedelund L., Moreau K.E., Beyer D.M., Nymann P., Haedersdal M. (2010). Fractional nonablative 1540-nm laser resurfacing of atrophic acne scars. A randomized controlled trial with blinded response evaluation. Lasers Med. Sci..

[15] Hession M.T., Graber E.M. (2015). Atrophic acne scarring: A review of treatment options. J. Clin. Aesthetic Dermatol..

[16] Alam M., Han S., Pongprutthipan M., Disphanurat W., Kakar R., Nodzenski M., Pace N., Kim N., Yoo S., Veledar E. (2014). Efficacy of a needling device for the treatment of acne scars: A randomized clinical trial. JAMA Dermatol..

[17] Yoshimura K., Sato K., Aoi N., Kurita M., Inoue K., Suga H., Eto H., Kato H., Hirohi T., Harii K. (2008). Cell-assisted lipotransfer for facial lipoatrophy: Efficacy of clinical use of adipose-derived stem cells. Dermatol. Surg..

[18] Jacovella P.F. (2008). Use of calcium hydroxylapatite (Radiesse) for facial augmentation. Clin. Interv. Aging.

[19] Guida S., Longhitano S., Spadafora M., Lazzarotto A., Farnetani F., Zerbinati N., Pellacani G., Galadari H. (2021). Hyperdiluted calcium hydroxylapatite for the treatment of skin laxity of the neck. Dermatol. Ther..

[20] Emer J., Sundaram H. (2013). Aesthetic applications of calcium hydroxylapatite volumizing filler: An evidence-based review and discussion of current concepts: (part 1 of 2). J. Drugs Dermatol..

[21] Braho K., Zevini A., Martinelli D., Barini R. (2025). Advanced Fractional CO_2_ Laser Treatment for Steroid-Induced Atrophy Scars: Clinical Outcomes. Am. J. Case Rep..

[22] Gennai A., Baldessin M., Melfa F., Bovani B., Camporese A., Claysset B., Colli M., Diaspro A., Russo R., Strano P. (2023). Guided Superficial Enhanced Fluid Fat Injection (SEFFI) Procedures for Facial Rejuvenation: An Italian Multicenter Retrospective Case Report. Clin. Pract..

[23] Melfa F., McCarthy A., Aguilera S.B., van Loghem J., Gennai A. (2024). Guided SEFFI and CaHA: A Retrospective Observational Study of an Innovative Protocol for Regenerative Aesthetics. J. Clin. Med..

[24] Melfa F., Gennai A., Carfagna G., Bovani B., Piccolo D., Colli M., Baldessin M., Siragusa D. (2023). Characterization of Adipose-Derived Mesenchymal Stem Cells from tissue harvested with the guided SEFFI technique and co-cultured with calcium hydroxyapatite. J. Appl. Cosmetol..

[25] Lesnik R.H., Mezick J.A., Capetola R., Kligman L.H. (1989). Topical all-trans-retinoic acid prevents corticosteroid-induced skin atrophy without abrogating the anti-inflammatory effect. J. Am. Acad. Dermatol..

[26] Pullar J.M., Carr A.C., Vissers M.C.M. (2017). The roles of vitamin C in skin health. Nutrients.

